# Cameras for Public Health Surveillance: A Methods Protocol for Crowdsourced Annotation of Point-of-Sale Photographs

**DOI:** 10.2196/resprot.3277

**Published:** 2014-04-09

**Authors:** Vinu Ilakkuvan, Michael Tacelosky, Keith C Ivey, Jennifer L Pearson, Jennifer Cantrell, Donna M Vallone, David B Abrams, Thomas R Kirchner

**Affiliations:** ^1^Department of Research and EvaluationLegacyWashington, DCUnited States; ^2^SurvosWashington, DCUnited States; ^3^Steven A Schroeder Institute for Tobacco Research and Policy StudiesLegacyWashington, DCUnited States; ^4^Department of Health, Behavior, and SocietyJohns Hopkins Bloomberg School of Public HealthBaltimore, MDUnited States; ^5^Department of OncologyLombardi Comprehensive Cancer CenterGeorgetown University Medical CenterWashington, DCUnited States

**Keywords:** image processing, crowdsourcing, annotation, public health, surveillance

## Abstract

**Background:**

Photographs are an effective way to collect detailed and objective information about the environment, particularly for public health surveillance. However, accurately and reliably annotating (ie, extracting information from) photographs remains difficult, a critical bottleneck inhibiting the use of photographs for systematic surveillance. The advent of distributed human computation (ie, crowdsourcing) platforms represents a veritable breakthrough, making it possible for the first time to accurately, quickly, and repeatedly annotate photos at relatively low cost.

**Objective:**

This paper describes a methods protocol, using photographs from point-of-sale surveillance studies in the field of tobacco control to demonstrate the development and testing of custom-built tools that can greatly enhance the quality of crowdsourced annotation.

**Methods:**

Enhancing the quality of crowdsourced photo annotation requires a number of approaches and tools. The crowdsourced photo annotation process is greatly simplified by decomposing the overall process into smaller tasks, which improves accuracy and speed and enables adaptive processing, in which irrelevant data is filtered out and more difficult targets receive increased scrutiny. Additionally, zoom tools enable users to see details within photographs and crop tools highlight where within an image a specific object of interest is found, generating a set of photographs that answer specific questions. Beyond such tools, optimizing the number of raters (ie, crowd size) for accuracy and reliability is an important facet of crowdsourced photo annotation. This can be determined in a systematic manner based on the difficulty of the task and the desired level of accuracy, using receiver operating characteristic (ROC) analyses. Usability tests of the zoom and crop tool suggest that these tools significantly improve annotation accuracy. The tests asked raters to extract data from photographs, not for the purposes of assessing the quality of that data, but rather to assess the usefulness of the tool. The proportion of individuals accurately identifying the presence of a specific advertisement was higher when provided with pictures of the product’s logo and an example of the ad, and even higher when also provided the zoom tool (χ^2^_2_=155.7, *P*<.001). Similarly, when provided cropped images, a significantly greater proportion of respondents accurately identified the presence of cigarette product ads (χ^2^_1_=75.14, *P*<.001), as well as reported being able to read prices (χ^2^_2_=227.6, *P*<.001). Comparing the results of crowdsourced photo-only assessments to traditional field survey data, an excellent level of correspondence was found, with area under the ROC curves produced by sensitivity analyses averaging over 0.95, requiring on average 10 to 15 crowdsourced raters to achieve values of over 0.90.

**Results:**

Further testing and improvement of these tools and processes is currently underway. This includes conducting systematic evaluations that crowdsource photograph annotation and methodically assess the quality of raters’ work.

**Conclusions:**

Overall, the combination of crowdsourcing technologies with tiered data flow and tools that enhance annotation quality represents a breakthrough solution to the problem of photograph annotation, vastly expanding opportunities for the use of photographs rich in public health and other data on a scale previously unimaginable.

## Introduction

Near universal penetration of mobile phones with built-in cameras marks the advent of a highly valuable platform for collecting data on the distribution of health-related features in the built environment. Photographs can provide detailed and objective information that cannot be detected by other surveillance and sensing systems. In addition, the ubiquity of mobile phones and ease-of-use of built-in cameras empowers citizens to participate in data collection on a scale previously unimaginable. Further enhancing the usefulness of photographs is the rapid advancement of the quality of geolocation services on mobile phones, allowing the linkage of mobile photographs to the physical location at which they were taken [1,2].

Photographs have long been recognized as a useful tool for public health advocacy and research, such as in the context of photovoice, a process in which individuals take photographs to relay their experiences with the goal of informing dialogue and ultimately enhancing their community [3]. More recently, photographs have dramatically improved resource evaluation and natural disaster response efforts around the globe [4,5], and in another recent example, smartphone cameras have been worn on lanyards by study participants to track individual health behaviors using life logging software that takes pictures at regular intervals [6].

It is important to note that photographs are not merely a method of validating traditional data collection modes such as surveys; rather, photographs are a rich source of data in and of themselves. In the context of surveillance research, collecting data in the form of photographs provides an interesting advantage over traditional surveys in that a set of questions need not be developed ahead of time. Instead, photographs can be taken of generic targets such as storefronts or street corners and later annotated to identify features of interest. This has significant implications for the need to hire and train skilled field workers for surveillance work, lowering the bar such that any citizen interested in collecting data about features of their environment that might impact the health of their community can take concrete action.

A logistical bottleneck that has previously inhibited the use of photographs for systematic surveillance has been the need to accurately and reliably annotate (ie, identify features within) large numbers of images. Technological advances enable photographs to be captured in large quantities, but the annotation of these photos is often an expensive and time-consuming process. It is seldom feasible to hire and train enough independent “raters” to examine photos and annotate them based on content, particularly when the number of photographs reaches in the thousands or more (multiplied, of course, by the complexity of information in each photo and the targets under study). The challenge is magnified when new questions requiring additional rounds of annotation arise, along with the ever-present need to address interrater reliability and the validity of the entire endeavor. For the first time, distributed human processing or crowdsourcing platforms make it possible to accurately, quickly, and repeatedly annotate photos at relatively low cost.

This paper describes a methods protocol for the crowdsourced annotation of photographs. Specifically, the paper outlines the development and initial testing of a set of custom-built tools that may greatly enhance the quality of crowdsourced annotation of photographs. The usability tests described in this paper were designed to assess the functional utility of the tools described, not to systematically evaluate them as part of an applied or experimental research study. Photographs were obtained from a research study on point-of-sale product categories and availability that took place from September 2011 to May 2013. During this time, two point-of-sale surveillance studies were conducted to better understand tobacco advertising in the retail environment, one in Washington, DC and one in New York City. In addition to photographs, trained field surveyors collected data through a variety of electronic methods, including phone-based interactive voice response, text messaging, global positioning system technologies, and a mobile application [1,7].

## Methods

### Crowdsourced Annotation Framework and Tools

Crowdsourcing is a relatively recent term, introduced in a 2006 *WIRED* magazine article and defined as an “open call to a large network of potential laborers” [8,9]. The 4 common categories of crowdsourcing include: (1) knowledge discovery and management, such as information gathering and organizing, (2) large-scale data analysis, such as photo coding and language translation, (3) innovation or problem solving contests, games, or platforms, and (4) generation and selection of the best idea [10]. This paper focuses on category 2, large-scale data analysis, namely the annotation of photographs. Photographs of the retail environment provide a good test case for crowdsourced annotation because they consist of complex scenes full of fine detail. This complexity enables the testing of different approaches and tools that might enhance the quality of crowdsourced photo annotation.

A number of studies have examined the use of crowdsourcing for purposes of image and photo annotation. Raschtchian et al [11] describe their experience using crowdsourcing to annotate images with multiple, one-sentence descriptions, and Sorokin and Forsyth [12] describe their experience crowdsourcing the annotation of images to outline any persons appearing in the image. Two more recent biomedical studies have used crowdsourcing to process images of biomarkers related to malaria and retinopathy [13,14]. In the field of public health, crowdsourcing has been used to aid in the annotation of images collected from webcams in order to identify active transportation [15], and in a system entitled PlateMate, which crowdsources nutritional analysis of photographs to determine food intake and composition estimates [16]. However, examples such as these are rare, and there is limited information in the literature regarding specific methodologies that can facilitate crowdsourced photo annotation, particularly tasks that require further processing of images (eg, zooming or cropping) in order to complete annotation accurately.

### Tiered Tasking Framework

Annotating photographs to the level of detail required for this project, which involves identifying the tobacco products, brands, and prices present in pictures of the point-of-sale environment, requires a tiered workflow. Decomposing the overall process into smaller tasks ensures that each separate task is simple, thereby improving the accuracy and speed with which the tasks can be completed and verified when needed. Tiered tasking also enables the simultaneous analysis of different photographs at different stages of the analytic process. Moreover, it enables the creation of an efficient workflow through adaptive processing, in which irrelevant or uninformative data are filtered out (eg, if there are no tobacco advertisements in a photograph, there is no need to ask for the identification of the type or brand of tobacco product advertised) and more difficult targets receive increased scrutiny (eg, if the crowd fails to reach consensus during the annotation of certain photographs, those photographs can be funneled to experts for further inspection).

Such a workflow greatly streamlines the crowdsourced photo annotation effort. For this project, an infrastructure was built to enable photographs to flow automatically from one stage of the process to the next. For example, in one workflow, photographs from a crowdsourced data-mining task (zooming into a storefront photograph and cropping out any tobacco advertisements) are automatically funneled into a subsequent annotation task involving identification of features, such as brand name or price.

Choosing a platform to operationalize this crowdsourced photo annotation workflow was the first consideration in building a customized annotation system. Amazon Mechanical Turk (MTurk) was the crowdsourcing platform used for this project. MTurk is a Web-based marketplace where “requestors” create open calls for “workers” (referred to as “raters” in the context of this project) to complete specified Human Intelligence Tasks (HITs) in return for a small payment. As such, raters completing crowdsourced tasks for the usability testing described in this paper are not selected or trained; they respond to an open call and the first raters to respond complete the task. MTurk is a marketplace for work that humans can achieve more effectively than computers, and image annotation is a prime example of such work [17]. While a simple interface for tasks is available within MTurk, for this project, MTurk application program interfaces were used to customize and configure workflow, task management, and reliability assessment processes external to the MTurk system. This enabled the automation of all processes, eliminating the need to export task results, manually filter data, and import back into the system, while still leveraging MTurk’s payment system and ability to attract workers.

### Photographic Zoom Tool

In deploying a task to identify the lowest advertised price for a cigarette within a photograph of a storefront, it quickly became apparent that it was difficult to identify and read individual advertisements and see details without a zoom capability.

**Figure 1:**
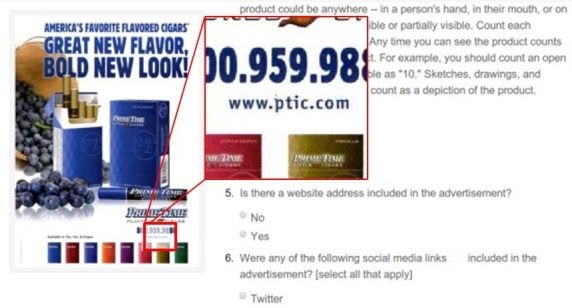
The Zoom Tool shows magnified detail while the mouse hovers over an image, temporarily obscuring the question.

There are various ways that a zoom tool can be incorporated within an MTurk task. One option is to configure the zoom tool in such a way that moving the mouse over a section on a photograph displays a close-up version of that section instead of the entire photograph. This would serve the need to see details more clearly, but users lose the ability to orient themselves within the context of the larger photograph. For the purposes of annotating storefront photographs, seeing the larger context of the photograph and where within it certain advertisements are situated can be very important. Thus, the zoom tool developed for this project was configured so that moving the mouse over a section of the photograph displays a close-up version of that section on a separate area of the screen, leaving the full photograph visible (Figure 1). Although this separate window blocks out a portion of the survey questions on the right-hand side of the screen while the user is zooming, the close-up image disappears once an answer to a question is found, and this process provides the critical advantage of being able to orient oneself within the overall context of the photograph.

### Zoom Tool Usability Test

A test was conducted to examine the usefulness of the zoom tool in improving annotation and to determine what other information in conjunction with the zoom tool might help equip raters in annotating a storefront photograph to identify specific tobacco advertisements.

This test involved 3 phases using a prototypical photograph of a storefront from prior surveillance work, in this case a storefront in the Central Harlem area of New York City. In phase 1, raters were asked if advertisements for Eon, Logic, and Blu e-cigarettes were present in the photo. In phase 2, using the same photo and questions from phase 1, raters were given additional assistance in the form of pictures of the typical point-of-sale advertisements for these e-cigarettes along with a logo for each of the e-cigarette brands. In phase 3, using the same photo and questions from phase 2, raters were also given the zoom tool in addition to the e-cigarette logo and advertisements to better help them identify the presence or absence of e-cigarette ads at the point-of-sale. Each of the three tasks was completed 100 times by 100 raters.

Raters who were equipped with additional imagery (brand logo and example advertisement, phase 2) or additional imagery plus the zoom tool (phase 3) were significantly more adept at identifying ads for Eon e-cigarettes than raters who were only provided a photo of the storefront (phase 1). When provided with a single storefront image, only 7.0% (7/100) of respondents were able to accurately identify the presence of an Eon advertisement. This proportion was significantly higher among respondents who were provided with pictures of the product’s logo and an example of the ad (73/100, 73.0%), and even higher among respondents given the zoom tool (88/100, 88.1%) (χ^2^_2_=155.7, *P*<.001). The Blu e-cigarette ad was more easily noticed in the original storefront image: 88.0% (88/100) were able to identify the ad looking at the storefront alone, with the proportion of respondents properly identifying ads being only slightly higher when provided with logo and ad images (92/100, 92.0%) and the zoom tool (92/100, 92.1%) (χ^2^_2_=3.70, *P*=.45).This task also contained a question about a Logic e-cigarette ad, which was not present on the storefront; adding additional information or the zoom tool did not improve accuracy (χ^2^_2_=6.89, *P*=.14).

### Photographic Crop Tool

The crop tool evolved from the need to indicate the exact location of a specific tobacco advertisement within the image of the storefront. Initially, a different tool was set up that could mark a single point on the photograph where the specific advertisement was found. However, there is no way to determine how large this point is relative to the rest of the ad. Capturing the close-up image from the zoom tool was considered as a way to identify the specific ad. The problem with this approach is that a fixed zoom size might not capture the boundary of the ad given that ads are different shapes and sizes. Thus, a slight variation of the zoom tool was created where a user could draw a box around what he or she wanted to zoom into, and then could press a button to capture that image. This is the custom-built crop tool used in this project.

Initially, the crop tool–related task was designed as a repeatable, multipart task. In this iteration, raters were asked to use the crop tool to draw a box around a single advertisement from an overall storefront photograph, and then answer questions about that cropped picture. Three questions were asked, beginning with asking the crowdsourced rater to identify the brand of the tobacco product being advertised. This question was formatted as either a dropdown (for short lists of brands, such as for e-cigarettes) or an auto-fill question (for longer lists of brands; auto-fill questions are similar to dropdowns, but filter results based on the initial letters typed). The second question asked about the price of the product advertised, and the third question asked about additional price-related information such as whether the advertisement includes information about a tax or special offer, if the price is per-pack or per-carton, and so on.

While this was a fast and efficient approach for experts attempting to complete the task, it was not well suited to crowdsourcing due to the difficulty of assessing reliability. In effect, a complex storefront photograph can be broken into dozens of pieces, each of which is associated with a different question, making it difficult to determine how similar responses are across raters.

As a result, the first modification of this tool and task was simplified to ask a single question, for instance, “draw a box around the lowest price for a pack of cigarettes you see in the picture”. The result for this question would be a set of coordinates describing the box. However, this approach only allowed for one picture per question, leading to a long list of repetitive questions.

**Figure 2:**
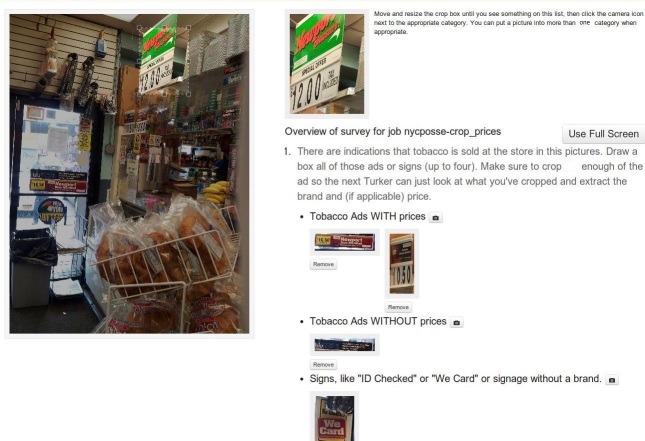
Example of a “tagged crop” task in which one or more selections within an image are associated with a tag, and multiple tags can be defined in a single question.

The next modification of this tool and task involved stringing together several smaller tasks, thus occupying a sweet spot between the expert-level, multipart crop task and the too-simple, basic crop that only answered a single question. In this version of the tool, called a “tagged crop” (Figure 2), multiple selections within an image can be associated with a tag. As illustrated in Figure 2, a single item can therefore produce a variable number of tags, each of which might be associated with multiple image selections.

This is a very efficient way to design a task and generate a set of images that answer specific questions. A simple task might ask raters to draw a picture around every tobacco ad in the storefront photograph, and categorize each as an advertisement with or without a price listed. A more complex task might have more options, asking raters to further separate ads and cigarette packs with shelf pricing, or asking them to separate out advertisements for different tobacco products (eg, combustible cigarettes, e-cigarettes, and little cigars).

Regardless of the way this crop tool is designed, one challenge that remains is that agreement on a cropped image is seldom exact; two raters may draw a very similar box and capture almost the same thing, yet their boxes are unlikely to be identical down to the level of pixels.

### “Black-Out” Reverse Crop Tool

Another remaining challenge is that in the absence of additional controls over data flow and processing, efficiency in annotating images can be seriously undermined by redundancy and inaccuracy due to false negatives (ie, failures to identify and crop a feature). Some easy-to-spot items might be identified many times, leading to redundancy, and other difficult-to-spot items might not be found by anyone, leading to inaccuracy. In order to address this issue, a “reverse crop” tool was conceptualized to further enhance the annotation process. The reverse crop tool removed the cropped section from the photo, leaving a background image with one or more rectangular areas that are blank or blacked out. This tool enables sections cropped by the first rater to be blacked out before the image is passed on to a second rater, so that the second rater is working from a less complex image, reducing redundancy and increasing the likelihood that no relevant advertisements are missed. An option to toggle on or off whether a rater can see the portion of the image that has been blacked out may be useful in cases where the initial crop is too wide and accidentally encroaches upon another portion of the image that could be a separate crop. This tool has the additional advantage of allowing a graded distribution of payment, with higher payments for crops of items that are more difficult to find.

### Crop Tool Usability Test

A test was conducted to examine the usefulness of cropping for annotating photographs of storefront advertisements. The test involved 2 phases. In the first phase, raters were presented with an unaltered storefront photograph and asked questions related to the presence of cigarette advertising and the identification of cigarette and menthol cigarette prices on the storefront. In the second phase, raters were presented with cropped advertisements from the overall storefront image and asked the same questions. In the first phase, there was one task, completed 100 times by 100 different raters. In the second phase, there were two tasks (one for each cropped image), and each task was completed 100 times by 104 different raters.

When provided cropped images, nearly all respondents (103/104, 99.5%) were able to accurately identify the presence of cigarette product ads on the storefront, a significantly greater proportion than among respondents provided only with an overall storefront image (65/100, 65.0%) (χ^2^_1_=75.14, *P*<.001). Respondents receiving cropped images properly identified which ads promoted menthol and nonmenthol product types (104/104, 100.0% reported that Newport menthol was a menthol product, and 103/104, 99.0% correctly reported Newport nonmenthol as an unmentholated brand).

Respondents who received cropped images of ads were significantly more successful at recognizing that prices appeared on the storefront (80/104, 76.5%), compared with respondents who were provided an image of the storefront alone (24/100, 23.5%) (χ^2^_2_=85.5, *P*<.001). Similarly, nearly all respondents who were provided with cropped images reported being able to read the prices (100/104, 96.6%), while none said they could read the prices when given the storefront image alone (χ^2^_2_=227.6, *P*<.001). When provided with a cropped image of Newport nonmenthol brand cigarettes, all respondents were able to accurately identify the lowest price in the image (mean $6.34, SD 0.00). For the Newport menthol image (accurate price, $7.29), the average identified lowest price was $7.24 (SD 0.45), which was not significantly different from the accurate price (*P*=.31). For respondents receiving only the storefront image, the lowest identified price was significantly less than the accurate price ($6.34), (mean $2.24, SD 0.72; *P*<.001). The storefront image contained an advertisement of a nontobacco product at a low price ($2.59), which respondents reported instead of the lowest-priced tobacco product. It is the only condition in which the average of lowest reported prices was significantly different from the accurate prices.

### Optimizing Crowd Size for Accuracy and Reliability

Data quality and reliability are of paramount importance when testing new data collection methods. Beyond improvements in feasibility and cost, a key advancement enabled by crowdsourcing is the potential to drastically improve methods for assessing interrater reliability. Reliability is improved not only because individual tasks can be simpler, but also because it is possible to collect large numbers of independent ratings. To capitalize on this methodology, a new “crowd-size resampling” approach to reliability assessment was developed.

Each MTurk task requires one to specify the desired number of raters, essentially defining the size of a simple random sample of raters drawn from the entire population of over 500,000 registered workers [18]. Because photos vary so much in quality and quantity of detail and, thus, coding difficulty, a very important challenge is that of determining the optimal number of raters needed to reach the best achievable level of accuracy regarding the contents of the photo. As is the case for any sample drawn from a population with unknown parameters, larger samples of raters will more closely approximate the actual population parameters as variance estimates decrease, while smaller samples will be more volatile. A researcher who selects a crowdsourced rater sample size that is too small risks unreliable results even when the interrater agreement is otherwise high, as smaller samples of raters will reach consensus about what is actually an incorrect answer more often than larger samples.

We sought to optimize crowd size by characterizing the degree of variation in accuracy among various sample sizes of raters in order to identify the point at which between-sample variation is stabilized, such that it approximated the true population level variance for a given task.

A population of 500 raters was established who all rated the same set of eight retail photographs along a number of dimensions. Jackknife resampling methods were then used to draw random subsamples of raters, beginning with 50 random samples of 2 raters, then 50 random samples of 3 raters, and so forth [19]. The ratings of each random sample of raters were evaluated relative to the ratings provided by trained field surveyors, making it possible to estimate the variance in accuracy derived from each randomly drawn sample of raters at each crowd size.

Receiver operating characteristic (ROC) regression was used to quantify the quality of this binary classification process, estimating the balance between true- and false-positive results from each photo rating task, relative to the results produced by our field surveyors [20,21]. A summary statistic called the area under the curve (AUC) was used to characterize the results from each ROC analysis. The AUC reflects the mean sensitivity, or true-positive fraction, averaged uniformly over the whole range of false positive fractions in 0,1 [20,21]. The resampling process produced pointwise CIs for the AUC at each crowd size, ultimately identifying the number of independent raters required to reliably approximate the maximum achievable AUC, given the characteristics of each given photograph and the difficulty of the question under study.

**Figure 3:**
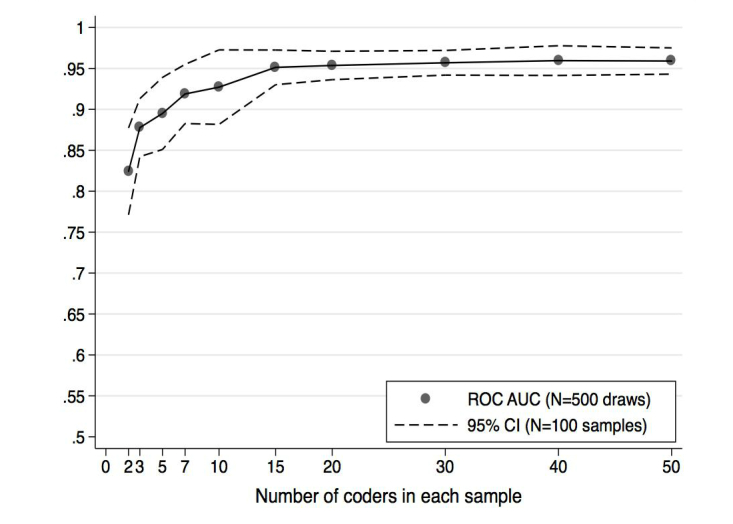
Area under the receiver-operating characteristic (ROC) curves produced by sensitivity analyses indicate an excellent level of correspondence between crowdsourced-rater and field-worker ratings.

Results reveal the optimal number of raters needed to compare the quality of crowd-sourced photo-only assessments with traditional field survey data collected at all DC tobacco outlets. Beginning with exterior store-front images, raters coded each for the presence versus absence of any tobacco product advertising, the presence versus absence of tobacco advertising that also included a price, and the presence versus absence of menthol tobacco product advertising. An excellent level of correspondence between crowdsourced-rater and field-worker ratings was found, with AUC produced by sensitivity analyses averaging over 0.95 (Figure 3). Crowd size needed to reach maximum correspondence ranged, however, with the more difficult menthol item requiring a considerable number of raters, 10 to 15 total, to get the variation of consensus above an AUC of 0.90.

## Results

Further testing and improvement of these tools and processes is currently underway. This includes conducting systematic evaluations that crowdsource photograph annotation and methodically assess the quality of raters’ work.

## Discussion

### Principal Findings

In the past, photos such as those described in this paper have remained uncollected or underused due to the extremely high burden of annotating images. Crowdsourcing makes it possible to access information contained within these rich data sources in a time-efficient and low-cost manner. This article describes an interface and associated methodologies used to crowdsource the annotation of photographs, including new approaches to improve the quality and reliability of this annotation.

Results from the zoom tool test suggest that the tool helps raters identify specific tobacco ads within a larger storefront photograph, particularly when paired with pictures of the product’s logo to aid in identification. Similarly, the test of the crop tool indicates that the tool improves raters’ ability to accurately identify the presence of cigarette product ads, as well as details about the ads, such as determining which ads are for menthol products and what tobacco product prices appear on a storefront. Finally, the ROC analysis confirms that in the context of photograph annotation, for more difficult coding targets, larger crowd sizes are likely needed to maximize the likelihood that results reflect the “emergent” accuracy available from the crowd. Methods such as the ROC analysis presented in this paper can be used in a preliminary step to estimate a reasonable crowd size to use for target types with different levels of difficulty.

Overall, results from testing the methods described here suggest that in the future, the traditional model of operationalizing manually collected field survey data as the gold standard for comparison should be flipped, instead collecting photographs to achieve coverage of an area and then surveying some proportion of the targets for the purpose of reliability. Interestingly, this photo archive can be integrated into a longitudinal record and mined for new information in perpetuity. This formative work demonstrates the scalable, sustainable nature of this solution, transforming an otherwise unfeasible task into one that is quite attainable and sustainable.

### Next Steps

Key next steps in this work include further testing and improvement of the tools and process described here, developing other tools and processes that will further enhance the quality of crowdsourced annotation, and conducting systematic studies that crowdsource photograph annotation and assess the quality of raters’ work in a methodical fashion. While the work described in this paper suggests that crowdsourced photograph annotation has promise, these next steps are necessary to systematically determine the quality of data produced through this process and how it compares with other traditional methods of analyzing surveillance photographs.

Further applications of crowdsourcing to public health research present a range of exciting opportunities. Advancements are needed to move beyond simple image tagging, in which images are tagged with certain identifying words, to extract more complex information, such as calculating the size of an object relative to others in the picture, examining change across a series of pictures, detecting anomalies in an image, and so on. This is an important area for development because questions such as these are challenging in the absence of computational assistance. For instance, questions about the size of objects or areas, and especially their relative size (eg, proportion of a store-front or lunch-tray left empty), are of great interest, but also of great difficulty to raters, to the point that reliable ratings have not yet been attained.

Leveraging crowd-sized scaling to optimize reliable and valid processing of data could prove more useful than anything else crowdsourcing has to offer public health researchers and advocates. In the past, manual processing of photographic data has often proven a cost-prohibitive or otherwise insurmountable challenge. Even when an effort is made to code images, there is pressure to burden raters with as many simultaneous coding tasks as possible to maximize the amount of data that can be obtained. In contrast, crowdsourcing enables the deconstruction of research questions into many smaller tasks, thus lowering the burden of any one task and minimizing difficulty and disagreement, subsequently increasing overall reliability. Crowdsourcing also offers the scale needed to conduct repeated independent ratings of images under study. As these methodologies iteratively improve, researchers will be able to adaptively optimize task design to maximize reliability.

Lastly, there are significant opportunities to go beyond annotation and expand the use of crowd-based systems for the collection of health relevant data itself. Researchers have already coined the term “participatory sensing” to describe the collection of data using mobile phones by individuals and communities; examples include exploration of transportation and consumption habits and reporting of problems and assets in the context of civic engagement and advocacy [22]. Combined with gaming dynamics like leader boards and scavenger hunts, the crowdsourced data collection approach will likely prove highly valuable as the need for information that can guide rapid response from regulatory and relief agencies continues to grow.

### Crowdsourcing Considerations

There are a number of important issues that must be considered when implementing a crowdsourced data processing project [10,23-25]. Most of these are beyond the scope of this paper, but we highlight a few that are directly relevant to the systems we have developed.

First are payment alternatives. MTurk’s built-in payment mechanism enables both flat-rate and bonus payments to raters, reducing the complexity and hassle of either building or using a separate third party payment mechanism. Researchers considering other crowdsourcing platforms should ensure a user-friendly payment system is either in place or can be created. Regardless, we recommend accounting for the hourly wage that can be reasonably obtained by any rater engaging with the task at hand, rather than simply focusing on minimizing costs. Providing the opportunity for raters to obtain reasonable compensation for their time and effort attracts and retains more highly skilled raters, and thus superior results.

Secondly, it is important to consider the reputation systems within a crowdsourcing community. The provision of accurate and detailed information about the quality of raters’ work has important implications for task design, especially in terms of how difficult a task can be. MTurk operationalizes reputation with a Worker HIT approval rate, which requestors can incorporate into task qualification requirements (eg, requiring that over 95% of prior HITs completed by the worker must have been accepted). The creation of more nuanced reputation systems across MTurk and other crowdsourcing platforms would likely enhance the quality of crowdsourced annotation. Reputations cut both ways, however, and research or advocacy groups must be aware of their own reputation within the crowd community when designing tasks and attempting to understand rates of completion, as well as quality and quantity of effort exerted by individual workers.

### Conclusions

Ultimately, there is great potential for the use of crowdsourcing for data collection and analysis in public health research, particularly data in the form of photographs. Iterative, in-depth experimentation with platforms, approaches, and tools is necessary to optimize the crowdsourced photo annotation process and explore further use of crowdsourcing for photo collection. As these tools are optimized, they will vastly increase capacity for large-scale, fast, high-quality photograph annotation, which in turn will expand opportunities for collection and analysis of photographs rich in public health data, and thus significantly advance our understanding of environmental influences on public health.

## Abbreviations

AUC: area under the curve
HIT: Human Intelligence Task
MTurk: Mechanical Turk
ROC: receiver operating characteristic
